# Moderating effects of personal innovativeness and driving experience on factors influencing adoption of BEVs in Malaysia: An integrated SEM–BSEM approach

**DOI:** 10.1016/j.heliyon.2021.e08072

**Published:** 2021-09-24

**Authors:** Hamed Khazaei, Mohammad Ali Tareq

**Affiliations:** Malaysia-Japan International Institute of Technology (MJIIT), University Technology Malaysia, Kuala Lumpur, Malaysia

**Keywords:** Electric vehicles, Technology acceptance, BEVs adoption, SEM, Structural equation modelling

## Abstract

Electric cars are relatively in the early stage of acceptance in Malaysia, the purpose of the current research was to determine the factors affecting the adoption of battery electric vehicles in Kuala Lumpur, Malaysia. This research utilized a quantitative method to gather and analyze the data and proposed a novel theoretical framework to explain the acceptance of battery electric vehicles. 500 surveys were distributed and 322 were gathered. Respondents of the study were University lecturers, postgraduate students, and employees in private companies. The results of SEM analysis indicated that the developed model provides a good fit for constructs used for this research. The result showed that social influence, facilitating conditions, environmental concern, and perceived enjoyment have positive effects on the adoption of BEVs. However, respondents indicated anxiety about the battery range. In conclusion, this study has contributed additional variables such as range anxiety and driving experience to the electric vehicle's acceptance literature. The findings are significant to electric car producers and policy makers who have environmental concerns to understand consumer prospective in this field.

## 1 Introduction

Governments are now looking for renewable resources and try to convince individuals to use cleaner energies (Anair and Mahmassani, 2012). Battery electric vehicles now have a direct impact on a cleaner environment, in countries with high contributions of renewable energies in electricity production (Lai et al., 2015). Although the usage of electric cars is one of the resolutions to decrease carbon dioxide production, their usage is still low in numerous countries including Malaysia (Khazaei and Khazaei, 2016). According to Anderson and Anderson (2010), for more than a century, the notion of electric vehicles has been of interest to many countries they are being more marketable worldwide. Carbon Dioxide (CO_2_) is one of the main greenhouse gases worldwide and specifically in Malaysia (Susskind et al., 2020). Electric Vehicles are introducing as a solution for environmental problems such as increasing the concentration of Carbon Dioxide and additional environmental issues. Mentioning the prominence of coping with global warming, numerous governments have started policies for reducing CO_2_ emissions by motivating, introducing, and production of electric cars. Because of several benefits of electric cars in energy saving and environmental protection, these vehicles without a doubt will have a great share of transportation in the future. The purpose of the current research was to determine the factors affecting the adoption of battery electric vehicles in Kuala Lumpur, Malaysia. The data was collected using a questionnaire, and the data were analyzed by applying an integrated SEM–BSEM Approach. The current study proposed a novel conceptual framework to explain and predict battery electric vehicle adoption. This study contributed to the literature on technology acceptability in the automobile environment by demonstrating the moderating effect of driving experience and personal innovativeness. The findings are significant for electric vehicle manufacturers and policymakers concerned about the environment, as they provide insight into customer attitudes on BEV usage. The sampling method used in this study aimed to survey postgraduates, University lecturers, and managers. Therefore, the sample of this study does not confirm that subdivision represents the entire population in Malaysia. Moreover, some of the respondents might not experience or even observed BEVs, and this might limit the strength of their responses. Therefore, the results of the study might not be generalizable for the whole Malaysian society.

## 2 Earlier studies on intention to buy hybrid and electric vehicles

Several studies have reported environmental benefits as drivers of BEV adoption intentions (Egbue and Long, 2012; Gallagher and Muehlegger, 2011; Graham-Rowe et al., 2012; Tamor et al., 2013). Beck et al. (2016) found environmental concern as one of the important factors that attract EV consumers. Peters and Dütschke (2014) studied Cost reduction policy, concerns about carbon emissions, and energy efficiency as drivers of consumer intentions to adopt BEVs. Hackbarth and Madlener (2016) argued that age, education, charging time, driving range, charging infrastructure, environmental effect, fuel cost, and policy have a significant impact on consumer intentions. Hidrue et al. (2011) studied the effects of driving range, purchasing cost, fuel cost, and charging time on intentions to adopt BEVs. With regards to charging vehicles at home, the results of Bühler et al. (2014) suggested enough family charging piles, driving interest, driving cost, low noise, purchasing cost, and driving range as important factors influencing their acceptance. Furthermore, they believed that providing charging facilities near the home can enhance customer adopting intentions. According to Zhang et al. (2011) government policies, the number of cars, and the number of family members that can drive are the factors affecting the adoption of a BEV. The battery range of BEV was found to be one of the major obstacles for customer adoption (i.e., Adepetu and Keshav, 2015; Barth et al., 2016; Dumortier et al., 2015; Egbue and Long, 2012). However, Jensen et al. (2013), argued that the adverse impact of limited range on customer intentions might be anticipated by their imprecise perceptions toward BEVs. The government policies, preferential tax, free parking, financial subsidies, and driving privileges were shown as factors that positively influence BEVs adoption (Hackbarth and Madlener, 2016; Helveston et al., 2015; Zhang et al., 2011). Burgess et al. (2013) found the practical experience as an essential factor in transforming the consumers' skepticism to a positive attitude toward acceptance. Driving a BEV helped users to develop more positive perceptions, particularly in terms of acceleration, speed, and low noise. Peters and Dütschke (2014) studied the influence of cost-reducing policy, carbon emissions, and energy efficiency on BEVs adoption. They also argued that customers with noble values have a propensity to choose their car considering environmental issues, while customers with egoistic values choose vehicles based on self-interests and personal benefits. Literature also recommended numerous individual traits including personal innovativeness, attitude, and self-efficacy instead of technology characteristics constructs (performance expectancy and effort expectancy) (Wisdom et al., 2014). Table 1 shows the summary of some earlier studies and the factors affecting buying behavior of hydrogen fuel, hybrids, and electric cars.

**Table 1:** Summary of previous studies on intention to buy hybrid and electric vehicles.

Author(s)	Factors	Technology
Aasness and Odeck (2015)	Financial incentives	Electric Vehicles
Adepetu and Keshav (2017)	Pro-environmental attitude, technology-oriented, purchasing cost, battery capacity, driving range	Electric Vehicles
Barth et al. (2016)	Driving range, purchasing cost, charging time, environmental effect, charging infrastructure, subjective social norm, collective efficacy, experience	Plug-In Electric Vehicles
Beck et al. (2016)	Government incentives, climate, experience driving range, energy crisis, Environmental concerns, vehicle emissions, driving habits, air quality	Plug-In Electric Vehicles
Bühler et al. (2014)	Driving interest, driving cost, low noise, purchasing cost, driving range	Electric Vehicles
Burgess et al. (2013)	Speed, sound, performance, a car of the future, look and style, the symbolic meaning of driving EV, environmental attributes, personal resistance, experience	Electric Vehicles
Cheron and Zins (1997)	Ease of use, range, performance, reliability, handling, low-cost, safety and price value of spare parts; Perceived risks: limited range, having an accident, mechanical failure, starting up issues, being stuck in traffic	Electric Vehicles
Carley et al. (2013)	Environmental Concern, appearance, Facilitating Condition, range, price value, charging time, car for the environment, innovation, independence on fossil fuel	Plug-In Electric Vehicles
Dumortier et al. (2015)	Driving range, Battery costs	Electric Vehicles
Egbue and Long (2012)	Technological level, driving range, environment effect, safety, charging infrastructure	Electric Vehicles
Gallagher and Muehlegger (2011)	Governmental tax incentives, Environmental Concern, fuel prices	Hybrid Vehicles
Graham-Rowe et al. (2012)	Cost minimization, vehicle adaptation demands, vehicle confidence, environmental Concern, impression management, and awareness of electric cars	Hydrogen Fuel Cell Vehicles (HFCV)
Hackbarth and Madlener (2013)	Fuel economy, driving range, charging infrastructure, emission reduction	Electric Vehicles
Hackbarth and Madlener (2016)	Charging time, driving range, charging infrastructure, environmental effect, fuel cost, government policy	Electric Vehicles
Helveston et al. (2015)	Subsidy policies, battery range	Electric Vehicles
Hoen and Koetse (2014)	Purchasing cost, fuel cost, total cost, financial benefit	Electric Vehicles
Hidrue et al. (2011)	Driving range, purchasing cost, fuel cost, charging time	Electric Vehicles
Jensen et al. (2013)	Purchasing value, range, carbon emissions, fuel costs, top speed, battery, stations, battery life, charging, environmental awareness	Full Electric Vehicles
Jensen et al. (2014)	Charging infrastructure, charging time, vehicle type	Electric Vehicles
Ko and Hahn (2013)	Policy incentives, charging infrastructure, swappable battery	Electric Vehicles
Klockner (2014)	Ascription of responsibility, awareness of need, social influence, descriptive norm, interjected norm, personal norm (Innovativeness), perceived behavioral control, awareness of consequences, attitude, intention	Full Electric Vehicles
Lai et al. (2015)	Economic benefits, high energy efficiency, policy, cheap electricity	Electric Vehicles
Lieven et al. (2011)	Buying price, range, car type, performance	Electric Vehicles
Noppers et al. (2014)	Functional attributes, environmental aspects	Electric Vehicles
Peters and Dütschke (2014)	Cost reducing policy, carbon emission, energy efficiency	Electric Vehicles
Pierre et al. (2011)	Charging time, domestic charging infrastructure, charging price, driving range	Electric Vehicles
Schuitema et al. (2013)	Instrumental, enjoyment, symbolic, pro-environmental identity, car-authority identity	Electric Vehicles (EVs)
Skippon (2014)	Dynamic performance, cruising performance	Hybrid and Full Electric Vehicles
Tamor et al. (2013)	Driving range, values, environmental concerns, charging infrastructures	Electric Vehicles
Tu & Yang. (2019)	Perceived Usefulness, Perceived ease of use, Compatibility, Personal Innovativeness, Interpersonal Influence, External Influence, Self-efficacy, Facilitating Conditions, Attitude Toward Behavior, Subjective Norm, Perceived Behavioral Control	Electric Vehicles
Xu et al. (2019)	Attitude, Perceived behavioral control, Subject norm, Environmental performance, Monetary incentive policy measures	Electric Vehicles
Vongurai. (2020)	Attitude, Brand Preference, Environment Concern, Fuel Efficiency, Social Influence	Electric Vehicles
Zhang et al. (2011)	Government policies, number of cars	Electric Vehicles

Since different demographical, individual traits, and psychological factors may influence different technology adoption, this study proposes a new conceptual framework by filling those theoretical gaps by adding range anxiety, driving experience personal innovativeness, and environmental concern in UTAUT 2 model to develop a model for predicting consumers acceptance in the context of BEVs.

**Figure 1:**
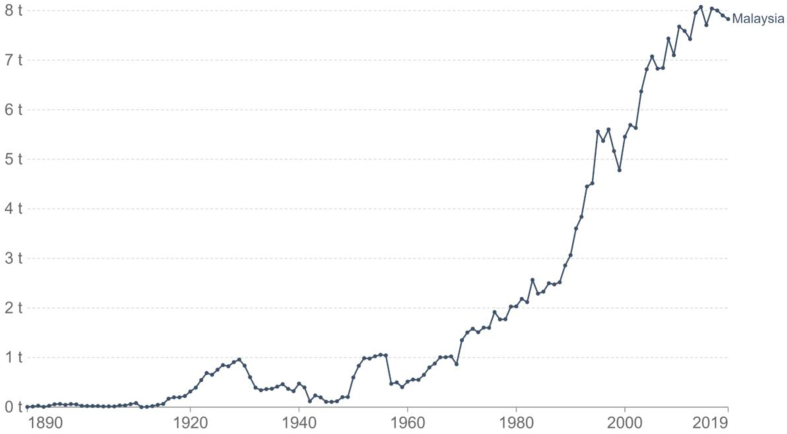
Cumulative CO_2_ production per capita from 1979 to 2019 (Ritchie and Roser, 2020).

In Malaysia, transportation is the main sector identified to produce carbon dioxide emissions (Sundram et al., 2021). By estimation, the amount of carbon dioxide produced in 2020, was more than 60 percent upsurge compared to the year 2000 (Nurgazina et al., 2021). There is no choice to reduce fossil fuel consumption and switch to harness renewable resources all around the world. Numerous countries counting Malaysia are now encouraged to reduce greenhouse gasses through international resolutions. Figure 1 shows the cumulative CO_2_ production from 1979 to 2019 (Ritchie and Roser, 2020).

To reduce Carbon Dioxide emissions, the Malaysian government established the Sustainable Energy Development Authority (SEDA). The aim was to promote the use of renewable energies in power plants, promoting public transportation, and inspiring the usage of sustainable technologies. According to the Ministry of Energy, Green Technology and Water the greenhouse gas (GHG) emission is projected to decrease by more than 40 percent by 2030 by the successful implementation of the Green Technology Master Plan (Susskind et al., 2020). In 2016 the Malaysian government announced its plan to promote battery electric vehicles in the country (Susskind et al., 2020). An essential advantage of BEVs is to improve the quality of life in terms of environment and health, which especially in urban areas will lead to sustainable transportation enhancement (Pita et al., 2020). Increasing the attention of both manufacturers and governments to electric vehicles is logical; however, widespread acceptance of electric cars come across several tough challenges. In Malaysia, public acceptance of BEVs as environmentally friendly cars was not satisfactory. Total sales for BEVs in Malaysia only accounted for less than 2% of total cars sold in 2019 (Markets Insider, 2020). Uncertain and limited range, together with long recharging time and insufficiency of charging stations might have a negative effect on the adoption of electric cars. The Malaysian authorities are now planning to increase the charging stations to 125,000 throughout the country by 2030, to overcome one of the important obstacles of BEV adoption (Suehiro and Purwanto, 2019). The hope however revived by deciding Malaysia to be a hub for the manufacture and supply of BEVs. Malaysian car manufacturers are now obligated to spend more than 200 billion US Dollars to develop BEVs and PHEVs hoping for further segment expansion in the future (New Straits Times, 2020). Rogers (2003) argues that one of the major causes for market failure of new technologies is resistance from potential consumers. An appropriate understanding of customer needs will help marketers to succeed in the market. To present a new product successfully, the wide-ranging needs and wants of the target market should be in focus (Niyonteze et al., 2020). Innovation resistance takes more than one form, and marketers should be aware of the range of situational and personality factors that could lead to resistance (Rogers, 2003). An appropriate understanding of customer needs will help marketers to succeed in the market. To present a new product successfully, the wide-ranging needs and wants of the target market should be in focus, and satisfying those determined necessities should be the foremost objective (Beliveau, 2012). One viewpoint on BEVs adoption is that the mass acceptance of electric cars is mainly dependent on consumers’ perception of them (Khazaei, 2019a, b). Accordingly, for increasing BEV adoption, it is significant to comprehend in what way customers remark BEVs and what are the barriers, against their adoption. It means it is crucial to comprehend the factors that influence the intention to use electric cars.

This study develops UTAUT 2 model by adding important factors influencing electric cars according to previous studies such as range anxiety, personal innovativeness, driving experience, and environmental concern. De Visser et al. (2010) found anxiety to have a significant effect on the decision-making process. Several studies addressed range anxiety as a significant determinant of BEVs (Curtis et al., 2010; De Visser et al., 2010; Axsen et al., 2015). Considering that electric cars are still in the initial stage of adoption in Malaysia consumer anxiety about using electric cars may be higher than in other countries. Rauh et al. (2015) investigated the relationship between driving experience and range anxiety toward EVs acceptance. The study showed that experienced drivers had less range anxiety on the cognitive and emotional level than inexperienced drivers. Moreover, electric cars are generally considered green technologies, so their acceptance behavior is measured as environmentally friendly conduct. Environmentally friendly behaviors are motivated based on a combination of self-interest and concern for society and the environment (Bamberg and Möser, 2007). Drivers are becoming more aware of environmental issues and broad public discussion on global warming due to carbon dioxide emissions, produced by cars, is more impacting buying decisions of consumers (Razak et al., 2014). Sevaral other studies were conducted to explore the environmental awareness of consumers, technology views, experiences, and interest in EVs (Bamberg and Möser, 2007). The results indicated that the sustainability and environmental benefits of electric cars have a major influence on EV adoption and a major potential barrier to widespread EV adoption is the uncertainty associated with electric cars' battery technology and sustainability of fuel source. Former scholars have found a significant relationship between environmental concern and intention to use new sustainable technologies (Moons and De Pelsmacker, 2012; Egbue and Long, 2012; Carley et al., 2013; Razak et al., 2014). Furthermore, according to the diffusion of innovation theory, innovativeness is a significant attribute of early adopters who have high pioneering characteristics (Rogers, 2003). Several other scholars confirmed the importance of personal innovativeness and the early adopters as they can facilitate the mass market acceptance of new technology (Jager et al., 2014; Morton et al., 2016).

According to the literature review (Table 2), no empirical research has studied the research constructs of this study in a single conceptual framework. Moreover, although the antecedent influence of driving experience and personal innovativeness has been studied in some investigations, very few investigations tested the moderation effect of these factors. In consistence with recommendations for further research and as researchers have not previously addressed those factors that affect the behavioral intention to use electric cars, the present literature gap will be addressed.

**Table 2:** Review of variables of this study in previous investigations.

Research Group	Source	Antecedent Factors					Moderator Factors	
		Social Influence	Facilitating Conditions	Range Anxiety	Perceived Enjoyment	Environmental Concern	Personal Innovativeness	Driving Experience
Electric Vehicles (BEVs)	Aasness and Odeck (2015)	-	-	-	-	-	-	-
Electric Vehicles (BEVs)	Adepetu and Keshav (2017)	-	-	✓	-	✓	-	-
Electric Vehicles (BEVs)	Barth et al. (2016)	✓	-	✓	-	✓	-	-
Electric Vehicles (BEVs)	Beck et al. (2016)	-	-	-	-	✓	-	-
Electric Vehicles (BEVs)	Bühler et al. (2014)	-	-	✓	-	-	-	-
Electric Vehicles (BEVs)	Burgess et al. (2013)	-	-	-	-	✓	-	-
Electric Vehicles (BEVs)	Cheron and Zins (1997)	✓	✓	-	✓	✓	-	-
Electric Vehicles (BEVs)	Carley et al. (2013)	-	✓	-	-	✓	-	-
Electric Vehicles (BEVs)	Dumortier et al. (2015)	-	-	✓	-	-	-	-
Electric Vehicles (BEVs)	Egbue and Long (2012)	-	-	✓	-	-	-	-
Hybrid Vehicles	Gallagher and Muehlegger (2011)	-	-	-	-	✓	-	-
Hydrogen Fuel Cell Vehicles (HECV)	Graham-Rowe et al. (2012),	-	-	-	-	✓	-	-
Electric Vehicles (BEVs)	Hackbarth and Madlener (2013)	-	-	✓	-	-	-	-
Electric Vehicles (BEVs)	Hackbarth and Madlener (2016)	-	-	✓	-	✓	-	-
Electric Vehicles (BEVs)	Helveston et al. (2015)	-	-	✓	-	-	-	-
Electric Vehicles (BEVs)	Hoen and Koetse (2014)	-	-	-	-	-	-	-
Electric Vehicles (BEVs)	Hidrue et al. (2011)	-	-	✓	-	-	-	-
Electric Vehicles (BEVs)	Jensen et al. (2013)	-	-	✓	-	✓	-	-
Electric Vehicles (BEVs)	Jensen et al. (2014)	-	-	-	-	-	-	-
Electric Vehicles (BEVs)	Ko and Hahn (2013)	-	-	-	-	-	-	-
Electric Vehicles (BEVs)	Klockner (2014)	✓	-	-	-	-	-	-
Electric Vehicles (BEVs)	Lai et al. (2015)	-	-	-	-	-	-	-
Electric Vehicles (BEVs)	Lieven et al. (2011)	-	-	✓	-	-	-	-
Hybrid Electric Vehicles and Full Electric Vehicles	Noppers et al. (2014)	-	-	-	-	✓	-	-
Electric Vehicles (BEVs)	Peters and Dütschke (2014)	-	-	-	-	-	-	-
Electric Vehicles (BEVs)	Pierre et al. (2011)	-	-	✓	-	-	-	-
Electric Vehicles (BEVs)	Schuitema et al. (2013)	-	-	-	-	-	-	-
Electric Vehicles (BEVs)	Skippon (2014)	-	-	-	-	-	-	-
Electric Vehicles (BEVs)	Tamor et al. (2013)	-	-	✓	-	✓	-	-
Electric Vehicles (BEVs)	Tu & Yang. (2019)	✓	✓	-	-	-	-	-
Electric Vehicles (BEVs)	Xu et al. (2019)	✓	-	-	-	-	-	-
Electric Vehicles (BEVs)	Zhang et al. (2011)	-	-	-	-	-	-	-

Ticehurst and Veal (2000) argue that culture affects the results of the study. Therefore, while those models or theories of technology adoption have been modified, established, and prolonged mostly in some countries, they still are useable in other countries or cultures. Consequently, evolving a model of technology acceptance in the Malaysian nation is significant and necessary for promoting the usage of this immature technology in this country. Overall, no empirical research has studied the research constructs of this study in a single conceptual framework in Malaysia. The research questions of this study are as follows:

### 2.1 Research questions

1. What influence does the social influence have on intention to use BEVs?
2. What influence does the facilitating condition have on intention to use BEVs?
3. What influence does the range anxiety have on intention to use BEVs?
4. What influence does the perceived enjoyment have on intention to use BEVs?
5. What influence does the environmental concern have on intention to use BEVs?
6. How does personal innovativeness moderate the relationship between social influences and intention to use BEVs?
7. How does driving experience moderate the relationship between social influences and intention to use BEVs?
8. How does driving experience moderate relationship between facilitating condition and intention to use BEVs?
9. How does driving experience moderate relationship between range anxiety and intention to use BEVs?

All the variables of the current research are being discussed as follows:

### 2.2 Intention to use

Individuals with the willingness to use new technology are those who made a conscious plan to use or buy that in the future (Moon, 2021). Intention to use has been proven to be one of the significant constructs in numerous technology acceptance theories (Venkatesh et al., 2003). The dependent variable “Intention to Use” was adapted from Venkatesh et al. (2012).

### 2.3 Social influence

Social influence is defined as the impact of society or peers' opinions on individuals' decisions to use an invention or new technology (Miao et al., 2016). The basic construct of social influence is a subjective norm from the theory of reasoned action and influences behavioral intention (Khazaei, 2020).

In Theory of Reasoned Action, Ajzen and Fishbein (1980) proposed the foundation of social influence construct as a predictor for specific behavior. Social influence as a direct determinant of behavioral intention is represented as the subjective norm in the Theory of planned behavior (Ajzen and Fishbein, 1980). The construct contains the explicit or implicit view that how an individual perceives others will view him/her because of using technology, will influence his or her intentions to use that technology (Venkatesh et al., 2012). Therefore, subjective norms or social influence is the social pressure applied on the individual or the decision-maker to perform the behavior (Miao et al., 2016). Khazaei (2016) suggested that the willingness of buying electric cars is influenced by the view of peers. Klockner (2014) recognized the psychological factors in different phases of the making decision process. Their results established the importance of both personal and social attributes that impact the decision to buy a vehicle. Yang and Chen (2021) argue that potential buyers of battery electric vehicles consider the influential and environmental characteristics of BEVs while considering social values. ‘‘They may not fully know or want to acknowledge that they buy and use sustainable innovations to show off or to feel good about themselves. Rather, people stress instrumental and environmental attributes of sustainable innovations’’ (Noppers et al., 2014). Consequently, examining how symbolic or social values affect the potential buyers in Malaysia is crucial as several studies have already stressed the significance and prominence of social norms among Malaysians (Onel, 2017; Hasbullah et al., 2016; Sin et al., 2012; Khazaei, 2020). Electric cars are often regarded as a status symbol, therefore, consumers' purchasing decisions will be restricted and influenced by the external environment such as the reference group and the social attributes (Wang et al., 2021b).

Therefore, the social standpoint about BEVs as new technology can raise the individuals’ attitude or intention toward purchasing them. According to the discussion above the first hypothesis is proposed:

H1 There is a significant association between social influence and intention to purchase BEVs in Malaysia.

### 2.4 Facilitating condition

Facilitating condition (FC) has been defined as the degree to which a potential customer believes that there is technological or organizational infrastructure exists for supporting the usage of the technology or product (Venkatesh et al., 2003).

This involves the perception of external and internal constraints on behavioral intention. FC is an independent factor that defines user's perception about ease of using the product (Venkatesh et al., 2012). In the context of BEVs, this could be construed as the availability of compatibility, spare parts, and charging infrastructures. This relationship is adapted from the extended UTAUT theory. Researchers suggested the facilitating conditions construct as a significant predictor of IT technology adoption (Zhong et al., 2021). Other studies also have found a meaningful correlation between facilitating conditions and intention to adopt new technologies (Venkatesh et al., 2012; Haris and Sugito, 2015; Maruping et al., 2017).

Prussi et al. (2021) argue that the retail infrastructure of the energies as fuel is crucial when introducing Alternative Transportation Fuels. This means that indicated vehicles using unconventional energy sources appeared to be competitive with conventional vehicles, by providing the fuelling infrastructures in place. Consistent with those arguments, Haustein et al. (2021) argued that refueling infrastructures will influence the decision-making process of considering buying a vehicle using electricity. They recommended fast charging infrastructures to facilitate long-range drives for BEVs, to increase market penetration of battery electric vehicles.

According to the above discussion second hypothesis is generated:

H2 There is a significant association between facilitating conditions and intention to purchase BEVs in Malaysia.

### 2.5 Anxiety

Anxiety about using technology is identified as of concern or emotional caution when it comes to using that technology (Yang and Forney, 2013). Anxiety, and attitude toward using technology, were not theorized to be direct factors of intention in the UTAUT model. From a theoretical viewpoint, this model shows that how the factors of behavior and intention evolved. The concern a person has toward the use of technology is stated as anxiety (Venkatesh et al., 2003). Compeau and Higgins (1995) introduced anxiety as a factor in the extended Social Cognitive Theory (SCT) in the context of computer use. De Visser et al. (2010) found anxiety to have a considerable impact on the decision-making process. Nevertheless, the correlation between anxiety and behavioral intention did not have a significant effect on behavioral intention in some studies (Venkatesh et al., 2003; Curtis et al., 2010). Considering that BEVs are still in the initial stage and their technology is not matured, buyer anxiety about using them possibly will be higher than anxiety about combustion engine cars.

### 2.6 Range anxiety

Range anxiety is described as apprehension about insufficient battery range of battery electric vehicles which may not reach the destination (Eberle and von Helmolt, 2010). Previous studies suggest that has found range anxiety as a negative predictor of the intention to purchase a limited range BEV (Egbue and Long, 2012; Luettringhaus and Nilsson, 2012; Nilsson, 2011; Dong et al., 2019; Franke and Krems, 2013; Axsen et al., 2015).

Herberz et al. (2021) argue that range, charging time, and battery issues were the factors hindering potential customers to use BEVs. Haustein and Jensen (2018) had a comparison of combustion engine and electric car users and found that perceived functional barrier as a hindrance of BEV adoption. Officials have invested a substantial amount of funds to decrease range anxiety among BEV customers (Lundström and Bogdan, 2012). Although the technology of batteries and BEVs are improving (Pagliaro and Meneguzzo, 2019) and ranges are increasing, but range anxiety is still a great concern of buying an EV and might have a negative direct influence on BEV acceptance and consumers may perceive risks when there is a lack of technology infrastructures like charging stations (Franke et al., 2012). As Result, the study is proposing the following hypothesis:

H3 There is a significant relationship between anxiety and intention to use BEVs.

### 2.7 perceived enjoyment

Wang et al. (2021a) regarded hedonic motivation as an essential foundation of motivation. A product perceived to have more pleasure to use is more likely to be adopted and develop more attitude toward using it (Miao et al., 2016).

Buyers' feelings and emotions have been shown to affect attitude and intention to use a product (Moons and De Pelsmacker, 2012). In their study, Moons and De Pelsmacker (2012) argued that potential buyer's perception about attractive emotions from driving an electric vehicle was significantly associated with customer attitude to adopt them. However, their research did not deliver additional information on the sort of consumer's positive emotions that expected to experience with BEVs. Schuitema et al. (2013) analyzed the role of feelings and perceived enjoyment including excitement, satisfaction pleasure, and pride. The findings showed that those participants who have more positive insights into the hedonic qualities of electric cars will have more positive feelings towards them, which in turn positively correlates to them to adoption (Schuitema et al., 2013). Miao et al. (2016) claim that the smoothness, acceleration, and engine performance of a car, all have a significant influence on the enjoyment of the driver and passengers. Another study argued that car customers are extremely interested in acceleration and power performance (Potoglou and Kanaroglou, 2007). Thananusak et al. (2017) also confirmed the value of higher speeding up the performance of vehicles when it comes to car buying intentions. Electric cars can achieve higher acceleration performance comparing with conventional vehicles in the same class, therefore the current study is investigating the role of enjoyment derived from higher performance to investigate if it can be considered as a predictor of intention to adopt BEVs.

Therefore:

H4 There is a significant correlation between perceived enjoyment and intention to use BEVs.

### 2.8 Environmental concern

Environmental concern has been discovered to be a considerable predictor of one's behavior (Weigel, 1983; Ajzen, 1991; SjÎberg, 1989; Takala, 1991; Moons and De Pelsmacker, 2012; Egbue and Long, 2012; Egbue & Long, 2012, 2012; Carley et al., 2013; Razak et al., 2014). Generally, BEVs are considered green technologies, so BEV acceptance behavior is measured as environmentally friendly behavior. Rafique and Town (2019) argues the higher effectiveness of BEVs would result in a reduction of carbon dioxide emissions even if all of them be charged using electricity from unsustainable resources. Environmentally friendly behaviors are driven based on a combination of self and society interest as well as the environment (Doszhanov and Ahmad, 2015). Broad public discussions about environmental issues due to carbon dioxide discharges by the transportation sector, specifically combustion engines, is now more influencing buying intentions of customers (Razak et al., 2014; Schuitema et al., 2013). Graham-Rowe et al. (2012) found that some consumers feel good driving electric cars use because of their associated environmental benefits. Because driving a “green car” allows people to show responsibility and a proactive role in society.

According to the above discussion fifth hypothesis is generated:

H5 There is a significant correlation between environmental concern and intention to purchase BEVs in Malaysia.

### 2.9 Moderating effects of personal innovativeness

The technology acceptance model did not include any moderator variable, but other studies including UTAUT and UTAUT2 models suggested moderators like voluntariness, experience, age, and gender into the basic TAM model to make a better explanation and examination of usage behavior of new technology. According to the diffusion of innovation theory, individuals’ innovative characters have a potential influence on the formation of their intentions to perform behaviors (Rogers, 2003). Innovators and early adopters are the leading individuals within marketplaces and they may have a degree of “thought leadership” for other potential consumers (Rogers, 2003).

Personal innovativeness is a characteristic feature of individuals related to their attitude towards new technologies of ideas (Khazaei, 2019a, b). An individual with high innovativeness makes their decisions to buy or use a new technology regardless of others’ opinion or experience (Jianlin and Qi, 2010). Personal innovativeness is argued to have a moderation impact on links among factors motivating behavioral intention (Alkawsi et al., 2021).

Considering electric vehicles, there are three motives for customer innovativeness which are hedonic, instrumental, and symbolic motives. Instrumental motives of the purchaser, stress the functionality of the vehicle. Perceived enjoyment focuses on the position of expected feelings, such as enjoyment from driving the vehicle. Symbolic innovativeness specifies the implication of symbolic aspects of the vehicle for the users (Schuitema et al., 2013; Morton et al., 2016). PI has bees theorized to have a moderation effect (Alkawsi et al., 2021; Cheng, 2014). According to the discussion above the sixth hypothesis is generated:

H6 Personal Innovativeness moderates the correlation between social influence and intention to use BEVs.

### 2.10 Moderating effects of driving experience

Experiencing a product suggested having a moderating effect on the correlation between social influence and intention to use that product (Venkatesh et al., 2012). Consequently, the impact of the social norm will decrease with increasing levels of experience (Venkatesh et al., 2003).

The findings of a study by Rauh et al. (2015) demonstrated that the driving experience of electric vehicles in practice was a significant factor for the driver to get information about how a BEV works and have a positive effect on their feelings and awareness of range dynamics. According to a study by Yang and Forney (2013), the experience of using a product makes the user more familiar with the technology involved and develops his/her knowledge about that product. In this study, respondents who drove a BEV once or more, have identified as experienced and were divided from respondents with no BEV driving experience. Venkatesh et al. (2012) suggested the moderation impact of the experience of product on the correlation between social influence and behavioral intention.

Thus:

H7 The driving experience of BEVs effectively moderates the association between social influence and intention of use of BEVs.

Experience also moderates the association between facilitating conditions and behavioral intention (Venkatesh et al., 2012).

H8 The driving experience of BEVs, moderates the association between facilitating condition and intention of use of BEVs.

Rauh et al. (2015) argue that experienced BEV drivers have less range anxiety on the cognitive and emotional level than drivers with no driving experience. Base on the above discussion, the ninth hypothesis is generated:

H9 The driving experience of BEVs, moderates the association between anxiety and intention of use of BEVs. In the current study, the driving experience was measured by a statement in the demographic section of the questionnaire using the following statement: “Have you drove an electric car before?”

## 3 Theoretical framework

The technology acceptance model is the development of the theory of reasoned action (Davis et al., 1989). This model used the theory of reasoned action as a theoretical foundation to propose the causal links among main constructs. Perceived usefulness and attitude are influencing behavioral intention. Perceived ease of use, perceived usefulness, users’ attitude, intention, and actual usage behavior while perceived usefulness and perceived ease of use are predictors of attitude (Davis et al., 1989). TAM replaced factors of attitude from the theory of reasoned action by perceived ease of use and perceived usefulness. Generally, the technology acceptance model specifies basic factors of technology acceptance and consequently has been applied to clarify or forecast individual behaviors through a wide range of technologies (Davis et al. 1989).

Davis et al. (1989) claimed that perceived ease of use would also affect perceived usefulness because when the usage of the system or technology is easier, then it will be more useful. The technology acceptance model assumes that using a specific technology is voluntary (Davis et al., 1989). The objective of the technology acceptance model is to describe the factors affecting technology acceptance that is overall capable of explaining user behavior across a wide-ranging sort of end-user technologies and user populations. However, since it combines findings gathered from many years of research, it might be particularly well matched for modeling technology acceptance (Davis et al., 1989). The UTAUT model was established as a complete incorporated model for better comprehending customer acceptance toward new technology or product. Based on Venkatesh et al. (2012), there are three ways that we can improve the estimate of technology adoption. First, they consider the customer acceptance of innovation in a selection of contexts such as population and culture. Next, they added diverse constructs to the model to extend the theoretic associations of UTAUT.

**Figure 2:**
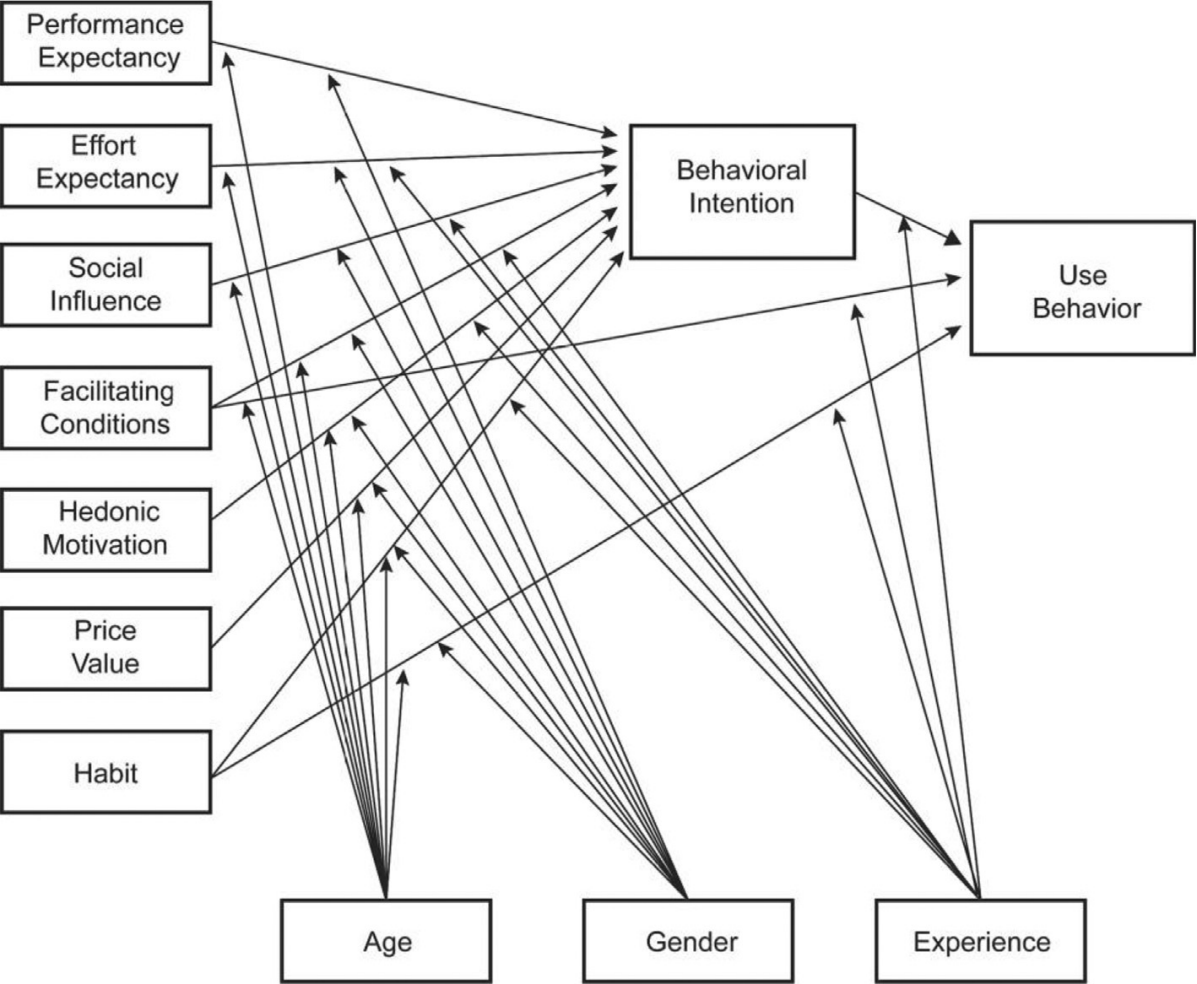
Extended unified theory of acceptance and use of technology (UTAUT2) (Venkatesh et al., 2012).

They reviewed several buyer behavior studies and amended their previous model to propose a new theoretical framework, which is UTAUT 2. Presently, this framework has progressively been implemented to explore the acceptance of various products. In this model, the perceived enjoyment or hedonic motivation factor was viewed as a significant predictor and was added to the UTAUT 2. The newly added constructs were confirmed repetitively in numerous studies as the important elements for users' technology acceptances (Figure 2 shows the theoretical framework of the UTAUT2 model). The previous model of the UTAUT has been used to define technology acceptance behavior in the organizational context. In its place, the model was extended from the UTAUT, and its focus is on individual perceptions in technology acceptance. UTAUT 2 significantly improved to explain variances in users’ technology acceptance.

Anxiety, self-efficacy, and attitude toward using technology, are not theorized to be direct factors of intention in this model. Gender, voluntariness, age, and experience have moderation effects in UTAUT. From a theoretical viewpoint, this model shows that how the factors of behavior and intention evolved. Venkatesh et al. (2003) also considered four moderators in UTAUT. Age, for instance, had very slight attention in the past research about technology acceptance. Nevertheless, the results of the UTAUT research specify that age moderates all the main associations in this model. Furthermore, gender is also a key moderator, which is consistent with the results of several studies (Venkatesh et al., 2012).

The UTAUT 2 is one of the latest acceptance models which consists of different determinants, which influence the overall user adoption of certain technologies. Since this study aimed to study the moderation effect of driving experience on BEV adoption, and the hedonic aspects of this product, the main advantage of using the UTAUT 2 model is that it has included these factors in the model itself. Moreover, since this model considered a wider range of variables, it is more suitable to be used for a product that is not in the information system technology field. The traditional TAM models were more often used to the adoption of technologies (computers, IT) at the workplace where an individual may not have the free will to deny those technologies. Although the UTAUT 2 model was well established as an incorporated model for better comprehending customer acceptance toward new technology or product, Venkatesh et al. (2012) supported the future research on their study to test their proposed model in the context of different technologies.

According to Venkatesh et al. (2012), there are three ways that estimation of technology adoption can be improved. First, they consider the customer acceptance of innovation in a selection of contexts such as population and culture. Next, they added diverse constructs to the model to extend the theoretic associations of UTAUT. The original UTAUT model may be reconsidered or modified for evaluation of more contextual factors that may explain adoption in different contexts suggested by Venkatesh et al. (2012). Some of the prior studies did not utilize some constructs of this model, especially moderators. For example, the usage of a certain IT technology might have been obligatory by the company but voluntariness, as a factor or moderator might not be relevant as the adoption of many technologies, are not obligatory (Wisdom et al., 2014). The current study did not consider voluntariness as the usage of BEVs is not compulsory.

This study evolved a technology acceptance framework through developing the UTAUT2 model to study the usage of the BEV technology in Malaysia. It is therefore projected that the framework with novel key findings in this study will be applicable for policy makers and car manufacturers and car dealers.

**Figure 3:**
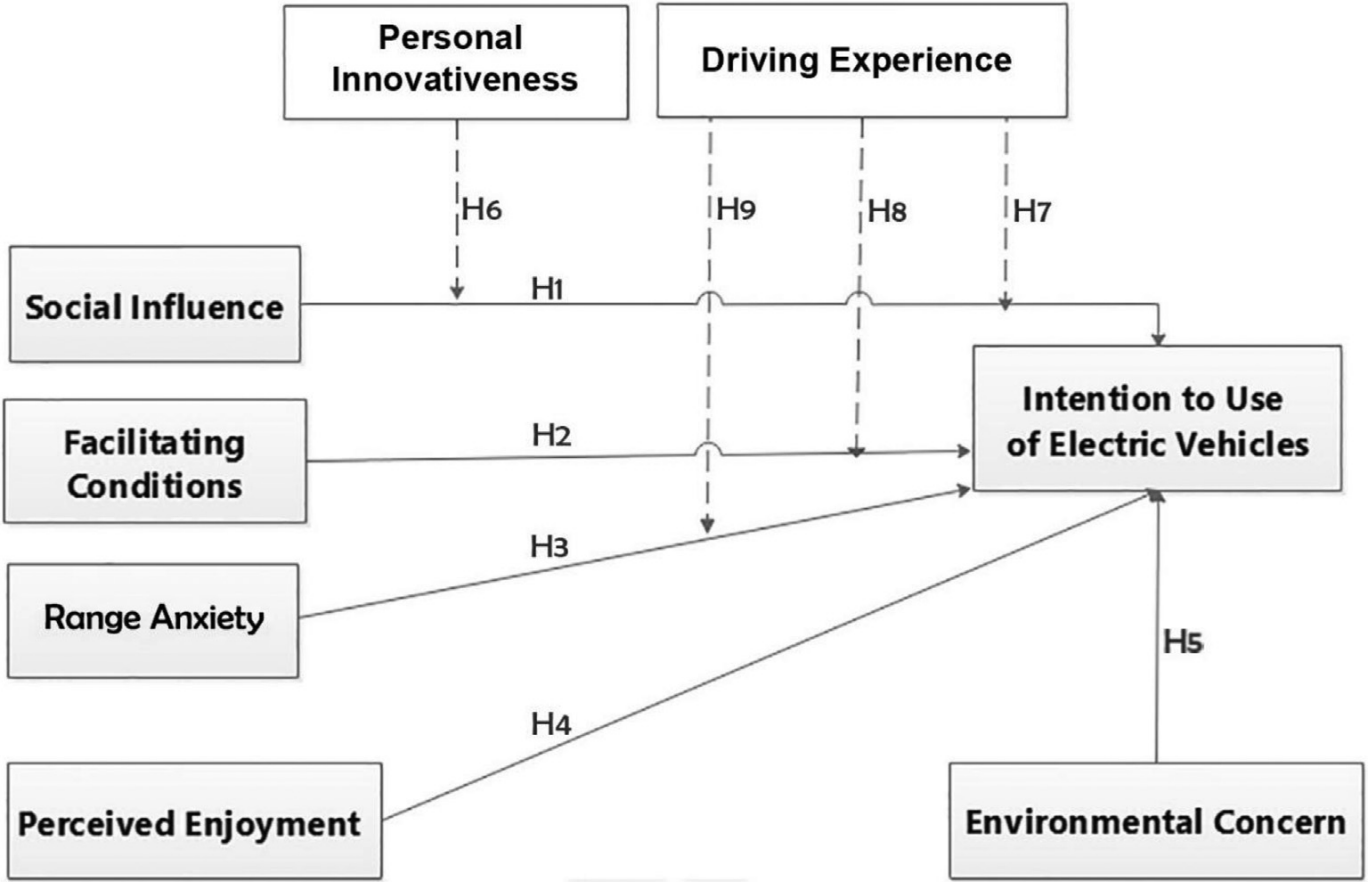
Conceptual model of the study.

Figure 3 illustrates the Conceptual Framework of the study.

## 4 Research instruments

This study used the Likert scale for designing questionnaires because of the advantages of this scale compared to other scales. Based on Johns (2010), the key advantage of Likert-Scale is that they are a well-known technique of collecting data, which made them easy to comprehend by respondents. It is straightforward to check for reliability and analyze the Likert scale in quantitative research. Moreover, respondents are free to give their opinions when answering the Likert Scale. And it is also easy to analyze the gathered data. Finally, it is swift and easy to complete this type of questionnaire and it can be sent out through all styles of communications, such as emails or even text messages (Johns, 2010). This study used a seven-point Likert scale for measuring study variables. Johns (2010) suggests that although having more scale points is better, yet there is a weakening return after around 11 points. Seven points scale is a decent choice without having too many response options while having enough points of discrimination (Johns, 2010). The survey consists of two sections. Section A includes demographic survey questions. Section B includes the measurement items of the study variables. The survey items related to the variables used in the study are presented and defined in Table 3:

**Table 3:** Measurement items.

Construct	Item	Source
Intention to use electric vehicles	INT1 If I had an electric car available, I would favor driving it rather than a traditional vehicle. INT2 If I were to purchase a vehicle within the next 5 years, I would purchase an electric car. INT3 I would recommend others to purchase an electric car. INT4 There is a high probability that my next vehicle will be an electric car.	Venkatesh et al. (2012)
Facilitating Condition	FC1 The resources necessary to use electric cars exist. FC2 I have the knowledge necessary to use electric cars. FC3 Electric vehicle is compatible with other technologies I use. FC4 I can get help from others when I have difficulties using electric car.	Venkatesh et al. (2012)
Social Influence	SI1 Driving a vehicle that attracts others' attention is important to me. SI2 An electric car would be a status symbol for me. SI3 Electric vehicles have a positive image in society. SI4 People react positively when they see an electric car on the road. SI5 People whose opinions are important to me find electric cars attractive. SI6 An electric vehicle would reflect my personality.	Venkatesh et al. (2012)
Perceived Enjoyment	PE1 Driving an electric car would be fun. PE2 Driving an electric car would be enjoyable. PE3 Because of smoothness and high acceleration, driving an electric car is very pleasurable for me.	Venkatesh et al. (2012)
Range Anxiety	ANX1 I have concerns about using electric cars. ANX2 The lack of enough infrastructure is somewhat frightening to me. ANX3 I be afraid that I may not reach my destination using an electric car. ANX4 I am afraid that I do not understand how to use the electric car.	Osswald et al. (2012)
Environmental Concern	EC1 I love to see a green environment. EC2 I want to preserve the environment. EC3 Electric car contributes to saving the environment for the next generation. EC4 Electric cars cause less pollution.	Razak et al. (2014)
Personal Innovativeness	PI1 If I heard about new technology, I would look for ways to experience it. PI2 Among my peers, I am usually the first to try out new technologies. PI3 In general, I do not hesitate to try out new technologies. PI4 I like to experience driving electric vehicles.	Agarwal and Prasad (1998)

## 5 Method

This research used a quantitative method to gather and analyze the data. 500 questionnaires were distributed, and 322 datasets with relevant responses were collected. The population of this study was those individuals who have knowledge about BEVs and may potentially be early adopters in Malaysia. Therefore, the researcher tried to survey those who know BEVs. To conclude, this study utilizes, and the quantitative method as the most suitable and appropriate approach for SEM (Hair et al., 2010).

This investigation used multiple analytic approaches that amalgamate Structural Equation Modelling (SEM) as well as Bayesian Structural Equation Modelling (BSEM) statistical procedures. Bayesian is a technique for estimating the probability density function of random variables with unknown parameters. Bayesian estimation is suggested for cross-validation of the results through ML estimation. Bayesian estimation unlike maximum likelihood (ML) estimation it does not rely on asymptotic theory (Kaplan, 2014; Byrne, 2010).

Assaf et al. (2018) also suggested the usage of Bayesian approach along with the SEM model that contains unobserved heterogeneities in a variety of random effects, to address some of the main limitations of the covariance-based method. However, an appealing feature of the Bayesian approach is that posterior distributions are obtained not only for the parameters but also for the latent variables (Muthén and Asparouhov, 2012).

Based on the above, a two-stage approach was be applied in this study. First, the SEM was employed to test the variables that have significant relationships with BEV purchase intention. Next, Bayesian estimation analysis was applied. Finally, the results of analysis based on both maximum likelihood (SEM) and Bayesian methodological estimates (BSEM) were compared to have an added advantage of parameter estimates accuracy (Byrne, 2010).

## 6 Sampling

Using (MLE) or Maximum likelihood estimation, with a sample size as small as fifty we can get valid results, but it is not recommended (Hair et al., 2010). A minimum of five participants per variable and not less than one hundred is suggested (Gorsuch, 1983). The research used the convenience sampling method.

According to the diffusion of innovation theory, innovators are the first consumers to buy and use new technology. They are comfortable taking risks and are enthusiastic about experiencing new ideas. Early adopters are followers of innovators in using new technologies (Rogers, 2003). Early adopters are commonly having a high level of education and income. Moreover, they have more access to scientific sources and interaction (Rogers, 2003). The current study tried to survey those who are possible early adopters of BEVs.

Therefore, the researchers stratified the entire population before employing random sampling methods, stratified random sampling correctly reflects the group under study. In a nutshell, it ensures that each subgroup within the population is adequately represented in the sample. As a result, stratified random sampling gives more comprehensive coverage of the population, as the researchers had control over the subgroups and can ensure that each is represented in the sampling.

Consequently, respondents of This study were university lecturers, and postgraduate students in the Kuala Lumpur campus of University Technology Malaysia (UTM), and employees of five enterprises in Kuala Lumpur, Malaysia. UTM. At the time of conducting this research, UTM Kuala Lumpur had 2420 postgraduate students and 366 lecturers. The researcher randomly distributed 210 questionnaires among postgraduate students, using Email and Google Forms. The researcher also randomly selected and contacted more than fifteen companies in various regions of Kuala Lumpur. Five enterprises agreed to participate in the study. 290 questionnaires were Emailed to managers and employees of the company to participate in the study. The data were obtained between August 2017 and May 2018 in Kuala Lumpur, Malaysia. 322 responses were gained, indicating a 64.4 percent response rate. The data obtained for this study is published and available in Data in Brief Journal (Khazaei, 2019a, b).

## 7 Missing data and outlier detection

Eight questionnaires were not completely answered, so those questionnaires were omitted and did not consider for further analysis. Data of the 314 remaining surveys were entered SPSS 22.0 and were cautiously examined for missing data. The Mahalanobis distance evaluation is used to detect outliers (Kline, 2005). The results of the Mahalanobis-D2 distance values are shown in Table 4.

**Table 4:** Mahalanobis D^2^ observations for outliers detection.

Mahalanobis-D^2^	Prob
19.86882	0.00053
19.40099	0.00066

Table 4 displays that two outliers were detected. To avoid statistical issues in the multivariate analysis, those outliers were deleted from the dataset and finally, 312 datasets remained to be examined (Hair et al., 2010).

## 8 Results

Table 5 illustrates the descriptive statistics for each variable. The standard deviation is a measure of the average distance between two values (Mishra et al., 2019). That is the degree to which data deviates from the mean. The standard deviations range between 1.226 to 1.568 implying that data points for all the variables are frequently close to the data set's mean (Mishra et al., 2019). The social influence had the highest mean with (4.69) while the lowest mean is facilitating condition with (4.19). The mean or average of a set of data is the central tendency of the data, i.e., the number around which the entire set of data is distributed. In certain ways, it is a single number that may be used to assess the whole value of the data set (Mishra et al., 2019).

**Table 5:** Descriptive statistics for all variables (n = 312).

	N	Min	Max	Mean	Std. Deviation
Intention to Use	312	1	7	4.44	1.526
Social Influence	312	1	7	4.69	1.413
Facilitating Condition	312	1	7	4.19	1.226
Anxiety of Use (Range Anxiety)	312	1	7	4.26	1.436
Environmental Concern	312	1	7	4.55	1.568
Perceived Enjoyment	312	1	7	4.48	1.560

### 8.1 Overall demographic profile

The frequency of descriptive analysis is shown in Table 6. About 71.2 percent of respondents were male and 28.8 percent were female. The major age group comprised of those aged 36–45 years old (36.5 percent), followed by the age group 46–55 years old (39.1 percent), then the age group over 55 years old (16.3 percent). About 14.7 percent were between 26 to 35 years, and one of the respondents was below 25 years old. The majority of the respondents were Malay (68.4 percent) followed by Chinese (18.2 percent).

**Table 6:** Demographic profile of respondents.

	Frequency	Percentage	Valid Percent	Cumulative Percent
**Gender**				
Female	90	28.8	28.8	19.5
Male	222	71.2	71.2	100
Total	312	100	100	

**Age**				
under 25	1	0.3	0.3	0.3
26–35	46	14.7	14.7	15.1
36–45	114	36.4	36.5	51.6
46–55	100	31.9	32.1	83.7
over 55	51	16.3	16.3	100
Total	312	99.7	100	

**Ethnicity**				
Malay	214	68.4	68.6	68.6
Chinese	57	18.2	18.2	86.9
Indian	27	8.6	8.7	95.5
Iranian	7	2.2	2.2	97.8
Other	7	2.2	2.2	100
Total	312	99.7	100	

**Education Background**				
Diploma/technical school certificate	26	8.3	8.3	8.3
Bachelor's degree or equivalent	59	18.9	18.9	27.2
Master's degree	102	32.7	32.7	59.9
Doctoral degree	105	33.7	33.7	93.6
Technical degree	20	6.4	6.4	100
Total	312	99.7	100	

**Monthly Personal Income**				
RM 2001–3000	24	7.7	7.7	7.7
RM 3001–4000	77	24.7	24.7	32.4
RM 4001–5000	95	30.4	30.4	62.8
Above RM 5000	116	37.2	37.2	100

**Do you have experience driving EVs?**				
No	272	87.2	87.2	
Yes	40	12.8	12.8	
Total	312	99.7	100	

### 8.2 Test of normality

The current study used skewness and kurtosis analysis for each variable to analyze the normality of the data using SPSS 22. The normality test shows that the values for skewness are between -1 and +1. Also, kurtosis for all items is between -2 and +2 (Hair et al., 2010). The results show a range of skewness between -0.0.024 to 0.244. Moreover, the kurtosis ranged from -1.368 to -0.319. Hence, it can be determined that the data set for all items are modeled well and distributed normally (Table 7).

**Table 7:** Normality analysis.

		Skewness	Kurtosis
inten1	312	-0.343	-0.951
inten2	312	-0.285	-0.748
inten3	312	-0.105	-0.685
inten4	312	-0.17	-1.001
soc1	312	-0.271	-0.691
soc2	312	-0.243	-0.681
soc3	312	-0.247	-0.787
soc4	312	-0.32	-1.001
soc5	312	-0.125	-1.274
soc6	312	-0.087	-1.368
fac1	312	0.244	-0.736
fac2	312	0.158	-1.193
fac3	312	-0.381	-0.789
fac4	312	-0.555	-0.532
anx1	312	-0.024	-1.057
anx2	312	-0.14	-0.917
anx3	312	-0.282	-0.855
anx4	312	-0.275	-0.841
env1	312	-0.591	-0.319
env2	312	-0.246	-0.914
env3	312	-0.249	-1.134
env4	312	-0.312	-0.853
pe1	312	-0.259	-0.957
pe2	312	-0.138	-1.078
pe3	312	-0.256	-0.818
pi1	312	-0.291	-0.917
pi2	312	-0.365	-0.998
pi3	312	-0.379	-1.005
pi4	312	-0.485	-0.817

To evaluate the extent of common method bias, Harman's one-factor test has been performed. The results showed no emergence of even a single factor as evidence for CMB existence, while the factor with the largest eigenvalues variance was 41 percent. Moreover, there was no indication of highly correlated factors (more than 0.95). Therefore, these evaluations show that there is no risk of measurement error due to common method bias according to (Kline, 2011).

#### 8.2.1 Convergent validity and reliability

Reliability in this study was measured by examining average variance extracted (AVE), Cronbach Alpha, and composite reliability (CR). Moreover, Table 8 shows that the AVE ranges from 0.521 to 0.895 and is more than 0.5 for all constructs as suggested by Kline (2011).

**Table 8:** Results of cronbach alpha and convergent validity.

Construct	Items	Internal Reliability Cronbach Alpha	Convergent validity		
			Factor Loading	Average Variance Extracted (AVE)	Composite Reliability (CR)
Intention to Use BEVs (INT)	4	0.887	0.870	0.755	0.925
			0.885		
			0.885		
			0.834		
Social Influence (SI)	6	0.759	0.722	0.521	0.867
			0.677		
			0.733		
			0.632		
			0.798		
			0.757		
Anxiety (ANX)	4	0.896	0.848	0.569	0.841
			0.901		
			0.905		
			0.840		
Facilitating Condition (FC)	4	0.739	0.747	0.764	0.928
			0.720		
			0.803		
			0.745		
Environmental Concern (EC)	4	0.786	**0.330**	0.636	0.863
			0.903		
			0.923		
			0.876		
Perceived Enjoyment (PE)	3	0.927	0.946	0.873	0.947
			0.953		
			0.903		
Personal Innovativeness (PI)	4	0.961	0.931	0.895	0.971
			0.961		
			0.964		
			0.927		

This study followed Hair et al. (2010) suggestion and applied the cut-off point of 0.7. After factor loading analysis, Therefore, two items from construct “Social influence” and one item from “environmental concern” were omitted. Values of composite reliability, for all constructs, ranges from 0.842 to 0.971, and all were greater than 0.7 which is suggested by Bagozzi et al. (1998). The Internal Reliability specifies how strong the measuring items are holding together in measuring the respective construct. All values of Cronbach's Alpha are more than 0.7 suggesting acceptable internal reliability (Bagozzi et al., 1998).

#### 8.2.2 Discriminant validity

Table 9 shows the discriminant validity of the constructs.

**Table 9:** Discriminant validity.

	FC	ANX	EC	SI	PE	INT	PE
FC	**0.874**						
ANX	-0.430	**0.754**					
EC	0.583	-0.547	**0.832**				
SI	0.596	-0.522	0.685	**0.722**			
PE	0.519	-0.522	0.763	0.664	**0.934**		
INT	0.559	-0.520	0.821	0.695	0.823	**0.869**	
PE	0.487	-0.471	0.700	0.598	0.664	0.720	**0.985**

Table 7 shows the correlations between different variables in the model are not surpassed 0.85 as endorsed by Kline (2011). Table 11 shows that the absolute correlation for each variable is lesser than the squared root of the average variance, demonstrating an acceptable discriminant validity among these constructs (Kline, 2011).

### 8.3 Indices of model fit

After data preparation, data were entered to SPSS AMOS version 24 for further analysis. The model is tested using Confirmatory Factor Analysis (CFA). Hair et al. (2010) suggested CFA as an appropriate method for researchers to confirm or reject a theory. Table 10 shows the values of the measures for the original model.

**Table 10:** Fit statistics of original measurement model.

Measures	X2	df	X2/df	GFI	RFI	IFI	TLI	CFI	RMSEA
Measurement Model	1295.622	237	5.467	0.731	0.762	0.827	0.797	0.826	0.120

**Figure 4:**
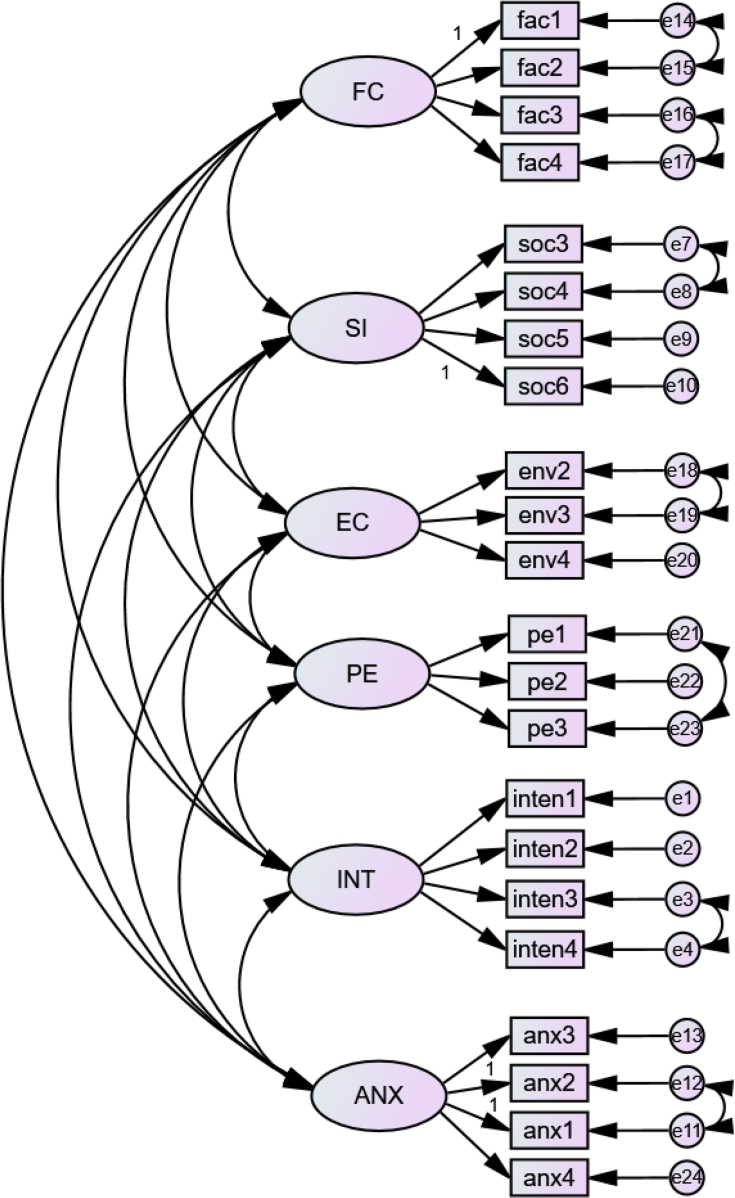
The measurement model after modification.

The model fit statistics suggest that the structural model is not suitable to represent a good fit to the data as suggested by Kline (2011), Byrne (2010), and Hair et al. (2010). Hence, modification is required. In the next process, the fit statistics for each construct were examined and modified separately. Figure 4 shows the measurement model after modification.

Table 11 presents the measure statistics for the measurement model with the modification. Evaluating the values shows almost all the model fit measures have better values after the modifications and these results show good overall model fit.

**Table 11:** Fit values for measurement model after modification.

Measures	X2	df	X2/df	GFI	RFI	IFI	TLI	CFI	RMSEA
Measurement Model	453.61	189	2.400	0.885	0.907	0.954	0.943	0.954	0.067

### 8.4 Path analysis

The Path analysis was used by the researcher to examine the impact of predictors on intention to use BEVs. AMOS calculates the critical ratio (C.R.), which is the coefficient divided by its standard error. Table 12 illustrates the Fit Values for the Structural Model. The results of path analysis are illustrated in Table 13.

**Table 12:** Fit values for the structural model.

Measures	X2	df	X2/df	GFI	RFI	IFI	TLI	CFI	RMSEA
Structural Model	520.338	189	2.753	0.885	0.894	0.943	0.930	0.943	0.075

**Table 13:** Regression weights.

Path			Unstandardized Estimate	Standard Error (S.E.)	(C.R.)	P Value	Standardized Estimate
INT	<==	EC	0.596	0.044	13.524	<0.01	0.674
INT	<==	PE	0.412	0.025	16.377	<0.01	0.628
INT	<==	ANX	-0.06	0.028	-2.026	0.034	-0.07
INT	<==	SI	0.196	0.025	7.984	<0.01	0.271
INT	<==	FC	0.234	0.071	3.308	<0.01	0.142

After four steps of modifications to the final adoption model, all remained variables have significant regression weight. All P values are less than 0.05 showing relationships are significant at a 0.05 significance level.

### 8.5 Moderation analysis

The model has two moderator variables to be tested. This study used SPSS 22 and Model 1 in the PROCESS macro of Hayes and Rockwood (2017) to analyze the moderation effects.

#### 8.5.1 Personal innovativeness (PI)

The results of moderation analysis for Personal Innovativeness (PI) are presented in Table 14.

**Table 14:** Moderator variable model (SI to INT).

Antecedent	Coefficient	SE	T	P-value
Constant	0.9755	0.5221	1.8683	0.0627
Social Influence	0.2208	0.1221	1.8082	0.0416
Innovativeness	0.2663	0.1146	2.3238	0.0208
Interaction	0.0480	0.0237	2.0306	0.0432

The results show that the P-value of interaction is 0.0432 which is less than 0.05 and the confidence interval does not include zero which indicates that personal innovativeness has a moderation effect on the association between social influence and intention. From the conditional effects of values of the moderator in Table 15, the moderation effect is significant in both lower and higher levels (Hayes and Rockwood, 2017).

**Table 15:** Conditional effects of personal innovativeness.

Innovativeness	Effect	SE	T	P-Value
2.7500	0.3529	0.0655	5.3916	0.0000
5.0000	0.4610	0.0434	10.6136	0.0000
6.5000	0.5331	0.0586	9.0934	0.0000

#### 8.5.2 Driving experience

The results of testing the moderation effect of driving experience on the association between social influence and intention are presented in Table 16. The results show that the P-value of interaction is 0.2943 which is more than 0.05 indicating that driving experience does not have a moderation effect on this relationship.

**Table 16:** Moderator variable model (SI to INT).

Antecedent	Coefficient	SE	T	P-value
Constant	4.4119	0.0626	70.5096	0.0000
Social Influence	0.7524	0.0455	16.5519	0.0000
Drive experience	0.6448	0.2351	2.7422	0.0065
Interaction	-0.1790	0.1704	-1.0505	0.2943

The moderation statistics of driving experience association among facilitating condition and intention to use BEVs is presented in Table 17.

**Table 17:** Moderator variable model (FC to INT).

Antecedent	Coefficient	SE	T	P-value
Constant	0.7130	0.1551	4.5984	0.0000
Environmental Concern	0.7995	0.0333	23.9827	0.0000
Driving exp	1.4986	0.6355	2.3581	0.0190
Interaction	-0.2108	0.1116	-1.8897	0.0597

The results show that the P-value of interaction is 0.0597 which is more than 0.05 and the confidence interval includes zero which indicates that driving experience is not a moderator on the correlation between facilitating condition and intention.

Table 18 shows the results consist of the causal effect of range anxiety on intention to use BEV and the moderation effect of driving experience.

**Table 18:** Moderator variable model (ANX to INT).

Antecedent	Coefficient	SE	T	P-value
Constant	4.5534	0.0856	53.2062	0.0000
Anxiety	-0.4874	0.0573	-8.5097	0.0000
Driving experience	1.4575	0.4121	3.5366	0.0005
Interaction	0.7537	0.2163	3.4853	0.0006

The results of Table 18 show that all coefficients are significant and most importantly the P-value of interaction is less than 0.001 and the confidence interval does not include zero which all indicate that driving experience has a moderation impact on the influence of range anxiety.

## 9 Stratified sample test

Due to gender imbalance in the sample of the study, and to address the concern of gender bias, the sample has been stratified based on gender. The researcher randomly omitted overrepresented male respondents and did the hypotheses test with the stratified balanced sample. Eventually, the process ended up with a gender-balanced sample of 184 respondents, including 94 males and 90 females. The results of Chi-square and hypotheses tests showed no difference between the stratified sample and the actual sample of the study. Table 19 shows the stratified sample demographic profile.

**Table 19:** Stratified sample demographic profile.

	Frequency	Percentage	Valid Percent	Cumulative Percent
**Gender**				
Male	94	51.1	51.1	51.1
Female	90	48.9	48.9	100.0
Total	184	100.0	100.0	

**Age**				
under 25	1	0.5	0.5	0.5
26–35	30	16.3	16.3	16.8
36–45	63	34.2	34.2	51.1
46–55	63	34.2	34.2	85.3
over 55	27	14.7	14.7	100.0
Total	184	100.0	100.0	

**Ethnicity**				
Malay	117	63.6	63.6	63.6
Chinese	44	23.9	23.9	87.5
Indian	17	9.2	9.2	96.7
Other	6	3.3	3.3	100.0
Total	184	100.0	100.0	
Malay	117	63.6	63.6	63.6

**Education Background**				
Secondary school certificate	19	10.3	10.3	10.3
Diploma/technical school certificate	17	9.2	9.2	19.6
Bachelor's degree or equivalent	80	43.5	43.5	63.0
Master's degree	56	30.4	30.4	93.5
Doctoral degree	12	6.5	6.5	100.0
Total	184	100.0	100.0	

**Monthly Personal Income**				
RM 2001–3000	18	9.8	9.8	9.8
RM 3001–4000	50	27.2	27.2	37.0
RM 4001–5000	49	26.6	26.6	63.6
Above RM 5000	67	36.4	36.4	100.0
Total	184	100.0	100.0	

The results of Table 20 show the results of stratified path analysis. Table 21 displays the results of the moderation analysis. The results show that there is no difference among the actual and stratified samples regarding the significant measurement weights, structural and moderation coefficients, suggesting that there is no statistically substantial difference among the results of the two models. Therefore, the results appear to be acceptable with the actual sample of the study.

**Table 20:** Stratified sample path analysis.

Path			Unstandardized Estimate	Standard Error (S.E.)	(C.R.)	P Value	Standardized Estimate
INT	<==	EC	0.606	0.055	11.012	<0.01	0.662
INT	<==	PE	0.388	0.035	11.224	<0.01	0.545
INT	<==	ANX	-0.061	0.035	-1.754	0.038	-0.076
INT	<==	SI	0.246	0.035	6.963	<0.01	0.311
INT	<==	FC	0.057	0.074	-0.766	0.041	0.043

**Table 21:** Moderator variable models for stratified sample.

Antecedent	Coefficient	SE	T	P-value
Constant	0.5473	0.6653	1.6323	0.1056
Social Influence	0.3456	0.3432	1.5559	0.0000
Innovativeness	0.1368	0.1096	2.9220	0.0208
Interaction	0.0480	0.0096	1.9996	0.0362
Constant	4.4119	0.1106	45.9376	0.0000
Social Influence	0.7524	0.2055	13.1676	0.0000
Driving experience	0.6448	0.4117	2.9902	0.0000
Interaction	-0.2543	0.2343	-1.2055	0.4675
Constant	0.6650	0.4919	4.4309	0.0000
Environmental C.	0.8432	0.1097	9.3067	0.0000
Driving experience	1.6458	0.4095	1.8031	0.0020
Interaction	-0.7108	0.1908	-1.2997	0.0906
Constant	3.6394	0.2354	8.6282	0.0000
Anxiety	-0.4556	0.1093	-5.9717	0.0000
Driving experience	2.1685	0.2181	3.1535	0.0000
Interaction	0.9003	0.4398	6.1093	0.0036

## 10 Bayesian structural equation modelling

Bayesian is a technique for estimating the probability density function of random variables with unknown parameters. Bayesian estimation is suggested for cross-validation of the results through ML estimation. Bayesian estimation unlike maximum likelihood (ML) estimation it does not rely on asymptotic theory (Kaplan, 2014; Byrne, 2010). However, an appealing feature of the Bayesian approach is that posterior distributions are obtained not only for the parameters but also for the latent variables (Muthén and Asparouhov, 2012). For small sample sizes, concerning the complexity of the model, estimations such as maximum likelihood often result in inadmissible, nonconvergence parameter solutions, and sometimes erroneous estimates. These concerns could be evaded by using Bayesian assessment (Kaplan, 2014). Bayesian estimation can be helpful to avoid some challenges, especially in the social sciences regarding gathering enough data, small populations, difficulties to contact target groups, or financial constraints (Smid et al., 2020).

In Bayesian SEM method, the values of the model parameters are assigned a joint distribution based on before the data are observed (prior distribution) and after being observed (posterior distribution) which will be combined during the process. This combined dissemination follows the Bayes’ theorem formula. Two key aspects of the joint distribution are the mean of the posterior distribution as the parameter estimate and the standard deviation of posterior distribution that serves as an analog to the standard error in Maximum Likelihood assessment (Byrne, 2010).

Therefore, conducting analysis based on both ML and Bayesian methodological estimates might have an added advantage for the researchers to compare the analysis of the parameter estimates (Byrne, 2010). Bayesian method describes and infers based on accurate posterior distributions of the latent variables and parameters. Bayesian SEM method is often suggested as a practical substitute for frequentist estimations, such as maximum likelihood estimation (Muthén and Asparouhov, 2012). Assaf, Tsionas, and Oh (2018) also suggested the usage of Bayesian approach along with the SEM model that contains unobserved heterogeneities in a variety of random effects, to address some of the main limitations of the covariance-based method. The Bayesian method involves the specifications of prior distributions for model unknowns comprising the parameters and the latent variables from the measurements and structural model (Kaplan, 2014). Moreover, Bayesian SEM model depends on the Markov chain Monte Carlo (MCMC) algorithm. MCMC suggests simulating draws from the combined posterior distribution of the model unknowns (latent variables and parameters) via a computationally rigorous process (Kaplan, 2014). Consequently, by utilizing Bayesian SEM and applying the MCMC procedure, the true values are diffused on the probability distribution (posterior distribution) to cope with the unknown occurrences. The benefit of this approach is that because accurate posterior distributions can be estimated for any function of the model unknowns, there is no requirement for relying on large sample assumptions. Those posteriors provide a more accurate measure of model uncertainty for small to moderate samples. This procedure reflects asymmetry and does not require the usage of other approximations or a delta method (Muthén and Asparouhov, 2012).

**Figure 5:**
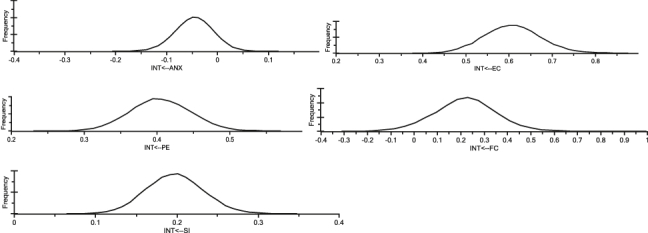
Posterior distributions of estimates.

In this paper, the Bayesian SEM analysis was performed by using IBM AMOS 24.0 software to assess the unstandardized weights produced by Bayesian approach and compared with the unstandardized estimates achieved in the analysis using the Maximum Likelihood SEM procedure. The posterior distributions via Markov chain Monte Carlo (MCMC) algorithm were disseminated and with the primary 500 burn in samples, took approximately 78,400 samples to allow the MCMC procedure to converge to the factual combined posterior distributions as C.S. values are less than 1.002 (Arbuckle, 2016). Figure 5 shows the posterior distributions of estimates Table 22 shows the Bayesian SEM statistics.

**Table 22:** Bayesian SEM statistics.

Regression weights	Mean	S.E.	S.D.	C.S.	Skewness	Kurtosis	Min	Max
INT<--EC	0.608	0.002	0.065	1.000	0.124	0.087	0.377	0.877
INT<--PE	0.405	0.001	0.041	1.000	0.040	-0.045	0.230	0.570
INT<--ANX	-0.045	0.001	0.038	1.000	-0.031	0.059	-0.207	0.120
INT<--SI	0.198	0.001	0.035	1.000	0.114	-0.016	0.064	0.348
INT<--FC	0.220	0.004	0.135	1.000	0.089	0.577	-0.310	0.996

**Figure 6:**
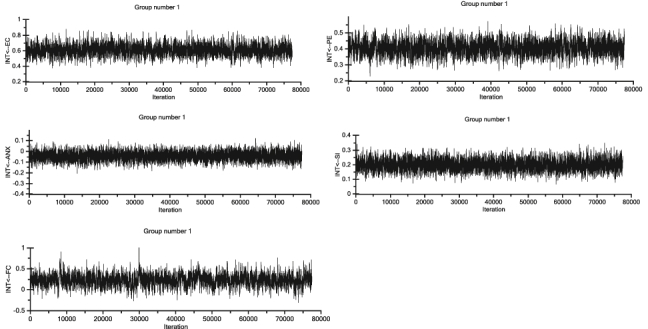
Traces of posterior mean values.

The estimations in Table 23 suggest that all parameter estimates (associations between constructs) are positive except for range anxiety which reinforces all the hypotheses. The S.E. showed that the parameter estimates of the posterior mean generated by MCMC were not too far from the true values of the posterior mean, implying the precision of the MCMC algorithm when generating analysis samples from the dataset (Kaplan and Depaoli, 2012). The value of the parameter estimates from the SEM appeared to be like the posterior mean of Bayesian SEM which validate the results of parameter maximum likelihood SEM estimations (Ong et al., 2018). Figure 6 exhibits the results of the trace plots and depicted the stability of the posterior mean values when the MCMC algorithm was generating analysis samples. The plots exhibit rapid up-and-down variation with no long-term trends or drifts. This indicates that the results of the Bayesian SEM were rather consistent and had no problems of reaching convergence (Arbuckle, 2016).

**Table 23:** Comparison between maximum likelihood (ML) and Bayesian estimates.

Causal Relationships Estimation			Bayesian SEM Estimates	Maximum Likelihood SEM Estimates
Intention	<==	Environmental Concern	0.608	0.596
Intention	<==	Perceived Enjoyment	0.405	0.412
Intention	<==	Anxiety	-0.045	-0.06
Intention	<==	Social Influence	0.198	0.196
Intention	<==	Facilitating Condition	0.220	0.234

Table 24 shows summary of hypotheses testing results.

**Table 24:** Hypotheses summary.

Research Hypothesis	Hypothesis	Results
H1	There is a significant relationship between social influence and intention to use electric cars.	Supported
H2	There is a significant relationship between facilitating condition and intention to use electric cars.	Supported
H3	There is a significant relationship between range anxiety and intention to use electric cars.	Supported
H4	There is a significant relationship between perceived enjoyment and intention to use electric cars.	Supported
H5	There is a significant relationship between facilitating condition and intention to use electric cars.	Supported
H6	Personal innovativeness moderates the relationship between social influence and intention of use of electric cars.	Supported
H7	Driving experience of electric cars, moderates the relationship between social influence and intention of use of electric cars.	Rejected
H8	Driving experience of electric cars, moderates the relationship between facilitating condition and intention of use of electric cars.	Rejected
H9	Driving experience of electric cars, moderates the relationship between anxiety and intention of use of electric cars.	Supported

## 11 Discussion

The current study proposed a novel conceptual framework to explain and predict battery electric vehicle adoption. Deriving from the UTAUT framework, this investigation theorizes that facilitating condition, range anxiety, environmental concerns, social influence, and hedonic motivation (perceived enjoyment) will influence the adoption of battery electric vehicles in Malaysia. The results of structural Equation Modelling suggested that the developed model provides a good fit of all the constructs used in the study. Moreover, the moderating effects of personal innovativeness and driving experience were also found to be significant in the recommended framework. Table 11 presents the impact of independent variables of the research framework on behavioral intention. Remarkably, environmental concern had the greatest effect on the intention to use BEVs with the regression weight of 0.674 (P-value < 0.01). Therefore, environmental concern is the most important predictor of BEV adoption in the model studied. Moreover, the results indicated perceived enjoyment as the second-highest predictor of BEVs (β = 0.628; P-value < 0.01). This implies the respondent's overall awareness of the performance of BEVs. This outcome also reveals the importance of the unique attributes of this vehicle which can be a considerable implication for car dealers. All the constructs of the study are discussed below:

### 11.1 Social influence

Empirical indication according to statistical analysis showed a significant and positive association between social influence and intention to use battery electric vehicles with a standardized Beta of 0.271. This result is in line with some previous studies. This result was observed in other studies (Venkatesh et al., 2003; Park et al., 2007; Mouakket, 2015; Wu and Chen, 2017). As a new vehicle becomes more apparent to others, it gains more public attention (Noppers et al., 2014). Potential adopters gain a better grasp of an invention by seeing it in action, which can result in higher adoption rates (Rogers, 2003). This is noteworthy and distinct from knowledge sharing within closely linked socioeconomic groups. Social norms can be descriptive (what other people do, what is usual) or prescriptive (what other people approve or disapprove of ideas about what is appropriate behavior for them). Information about referent social groups' rules and standards of behavior might lead to imitation or compliance. The findings suggest that as more evidence of the significance of social approval on people's adoption of BEVs in Malaysia. Potential adopters of new technology receive certain signals or social cues from others, which are especially powerful when they originate from referent or aspirational groups like those with comparable income levels or opinion leaders who promote innovations into a social network. This is a confirmation of the results of a study by Aini et al. (2013). They revealed that compliance with societal standards explained almost 12% of the variance in Toyota Prius purchasing decisions in Malaysian society. Schuitema et al. (2013) also argue that buyers of luxury goods, whose vehicle selections send messages to others about their social standing and identity, are particularly interested in signals from referent social groups.

In the United States, Axsen and Kurani (2012) found that the social symbol of electric cars affects the purchase intention of families. The respondents regarded BEVs as a sign of distinction that expresses their characters such as maturity, and intelligence.

In China, Zhang et al. (2011) studied 299 participants from different driving schools in Nanjing. The findings indicated that the acceptance of using battery electric vehicles is influenced by the view of colleagues and society. The Value Belief Norm (VBN) theory describes how the environment and society influence behaviors (Bamberg and Möser, 2007). BEV users do care about the people's opinions especially the ones that are in the higher levels of education, income, or fame in the society. From the sociological perspective, activities and maintaining a given behavior is not only personal but also social. People implement a certain behavior if it is commonly considered to be reasonable. Consistent with prior investigations on the importance of social norms, the results of this study showed most of the respondents will regularly adjust their behavioral intentions in line with society. The others' behavior and the way they think about the people are important for the potential BEV users in Malaysia and this will affect their buying intention. Society can encourage people to use the products. Therefore, if society accepts the new technologies, it will encourage the potential users to accept and adapt to a new product. Introducing electric vehicles to the public indicating their benefits by using a variety of media will be helpful. Also, persuading the managers and top managers of the organizations to use electric vehicles can result from the better adoption of the other users.

### 11.2 Facilitating condition

The results of statistical analysis indicated a significant and positive association among facilitating conditions and the intention to adopt electric vehicles (β = 0.142, p < 0.01).

Facilitating conditions have been proven to statistically significantly affect the behavioral intention to use m-shopping fashion apps, which is consistent with previous work done by Chong (2013), Venkatesh et al. (2012). This implies that consumers find it important to have the necessary support and help while using m-shopping fashion apps, and the more support, and guidelines they have the more they are willing to use m-shopping fashion apps.

This outcome showed that potential purchasers believe that the number of charging stations for battery electric vehicles is insufficient in Malaysia and that expanding that will influence their purchase intention. The results suggested that individuals are likely to purchase electric vehicles when they believe that there are enough technical or governmental infrastructures that support their usage of BEV. Hence, their view linked with their acceptance is reflected in their perceptions about the existence of technical support. Therefore, expanding supporting facilities will enhance the adoption of BEVs. The policies that governments can consider boosting BEV demand besides fiscal incentives are expanding charging grid, special lane access, charging infrastructure, toll exemptions and access to low emission zones can also be considered. Then the BEV users may experience a flawless trip knowing that there are accessible charging stations along the way, rather than having to continually monitor their driving pace and worry about draining the battery before reaching their destination.

This result is consistent with previous studies. Numerous studies suggest facilitating conditions as a predictor of new technology adoption (Venkatesh et al., 2003; Carley et al., 2013; Montano and Kasprzyk, 2015; Maruping et al., 2017). Taylor et al. (2010) identified the barriers of electric vehicle purchase intention: absence of a variety of choices; lack of charging infrastructure; and the potential increase in electricity rates. Tan et al. (2014) argue that buying behavior is influenced by factors such as charging issues, range anxiety, mental factors, and cost. Bockarjova et al. (2014) suggested the necessity in improving charging infrastructure as charging difficulty comparing with a conventional car is a significant barrier for EV adoption in Netherland. An investigation by Zhang et al. (2011), found these factors to influence electric cars adoption: insufficient EV incentives, local protectionism, uncertain EV market, and incompatible charging infrastructure in China. Carley et al. (2013) claimed that price; charging facility and charging time are also hurdling purchasing an electric car in the U.S.A.

The result also confirms that the respondents had the awareness about battery electric vehicles and that aspect qualifies individuals to understand the need for infrastructures like availability of charging stations, obtainability of spare parts, and compatible charging plugs to support their usage of electric vehicles. Consequently, the charging time and availability of distribution grids are essential for policymakers, scientists, and producers to enhance since it improves the tactics to popularize and promote BEVs. In conclusion, it is recommended that the government conduct a pilot design of charging stations in different areas of Kuala Lumpur or other populous cities, such as shopping malls, by encouraging investments from applicable producers through subsidies, to alleviate the obstacle of charging BEVs in Malaysia.

### 11.3 Range anxiety

As expected, range anxiety significantly and negatively impacted the individuals' intention to use electric vehicles. (β = -0.07, p < 0.05). Range anxiety influences by individuals' emotions, which decreases the propensity to accept electric vehicles. Individuals with a high degree of range anxiety tend to reject electric vehicles. This research shows that user's optimistic view of electric vehicles escalates when they feel secure driving and be sure of enough battery ranges and enough charging stations. Having a spare full battery that can be easily changed can help decrease their skepticism. By remarking on the usefulness of electric vehicles and having a positive perception about them, makes people readier to use them.

Considerable resources need to be invested in finding ways to reduce range anxiety in EV users (Lundström and Bogdan, 2012). Although the technology of batteries and EVs are improving and ranges are increasing, but range anxiety is still a great concern of buying an EV and might have a negative direct effect on EV adoption. The increase of traveled distance between charging events over the consumers may perceive risks when there is a lack of technology infrastructures like charging stations (Franke et al., 2012). Range anxiety was a barrier to EVs adoption, several studies have inferred that people are not willing to choose EVs due to range anxiety and inconvenient recharging access (Egbue and Long, 2012; Luettringhaus and Nilsson, 2012; Nilsson, 2011; Axsen et al., 2015). Franke and Krems (2013) have found that range anxiety is a negative predictor of the intention to purchase a limited range EV. Range satisfaction (Franke and Krems, 2013) and users’ confidence for using the EV for longer trips (Carroll and Walsh, 2010).

De Visser et al. (2010) found anxiety to have a significant effect on the decision-making process. Nevertheless, the correlation between anxiety and behavioral intention did not have a significant effect on intention in some studies (Venkatesh et al., 2003; Curtis et al., 2010; Bailey, 2017). These findings are consistent with (Carroll and Walsh, 2010; Nilsson, 2011; Egbue and Long, 2012; Luettringhaus and Nilsson, 2012; Franke and Krems, 2013). Based on the discussion above, governments should improve strategies that decrease the buyer's anxiousness, which leads to increase purchasing intention of electric vehicles. The significant negative effect of range anxiety on behavioral intention has theoretical and practical implications as very few studies considered this construct in the technology acceptance model.

This study integrated the hindrance factor “range anxiety” in the research framework. The item has been proved to be one of the major rationalizations delaying consumer decision to buy BEVs, yet very few prior studies have incorporated this construct in an adoption model such as UTAUT2 to predict purchasing BEVs.

### 11.4 Perceived enjoyment

This study indicated a significant positive effect of perceived enjoyment on electric vehicle adoption (β = 0.628, p < 0.01). Accordingly, hypothesis four is supported which is in line with other studies in the technology acceptance area. (Potoglou and Kanaroglou, 2007; Agrebi and Jallais, 2015; Rezvani et al., 2018).

This result shows that car use and ownership are frequently related to instrumental and hedonic characteristics. Perceived Enjoyment has been explained as the willingness to initiate acts that enhance the positive pleasurable experience while reducing the bad experience in terms of fundamental human experience and behavior (Liao et al., 2008). Individuals enrich their subjective well-being, initiate, and continue productive behaviors based on hedonic pursuit. This outcome confirms that low noise, smoothness, and high acceleration drives satisfaction for drivers and owners of battery electric vehicles. This is because BEVs are endowed with technological breakthroughs and much fewer mechanical systems as opposed to combustion engine cars. Potoglou and Kanaroglou's, (2007) stated that potential customers extremely value high maximum speed and acceleration performance. Therefore, this finding can be supported by the point that electric vehicles are smoother, with high torque and acceleration. The reputation of high acceleration performance based on electric vehicles is stated in another survey about vehicle buying intention. They argued based on their study that regardless of buyer's income, they are eager to pay a premium price for higher acceleration performance.

An investigation in China about people's intention to buy hybrid electric cars argues that a customer considers the cost of the car itself and operations such as battery range and maximum speed (Liu and Santos, 2015).

Miao et al. (2016) claim exterior and interior of a car, smoothness, acceleration, and engine performance, all are influencing the satisfaction of both driver and passenger. Customers extremely value acceleration performance and high maximum speed (Potoglou and Kanaroglou's, 2007). Lin and Wu (2018) also supported the significance of acceleration performance and power as unique advantages of EVs in their study concerning car purchasing in China. Therefore, while factors like maximum speed, size of the vehicle, and fuel economy were found less important, individuals with both low and high income stated that they would be willing to pay a price premium for high acceleration performance (Lin and Wu, 2018). Schuitema et al. (2013) suggests that performance factors are significant for the acceptance of the adoption of electric cars. Electric cars can achieve much more high acceleration performance comparing with conventional vehicles in the same class, it can be considered as perceived enjoyment for customers of electric cars. Consequently, consistent with the prior studies on hedonic and emotional attributes which influence consumers' behavior, this study confirms that the high acceleration performance of BEVs can improve their image in the views of potential consumers. More interestingly this attribute can even compensate for the issue of long recharging times, lower range, and greater costs (Noppers Klockner et al., 2013). As a result, perceived enjoyment can be considered a source of motivation to buy BEVs.

### 11.5 Environmental concern

According to the results of data analysis in this study, environmental concern was found to have the biggest significant positive effect on BEV adoption (β = 0.674, P < 0.01). According to this outcome, environmental concerns and public perceptions of environmental policy is an important element influencing customer attitudes toward BEVs, which subsequently influence purchasing or behavioral intentions. Aside from fuel savings, and great energy efficiency, car owners seek environmental benefits. Environmental concern is the evaluative response towards environmental issues. Thus, governments and car dealers are essentially required to realize that the users are becoming more environmentally friendly. Consequently, in line with earlier investigations, this finding convinces that degree of environmental concern may have a direct and strong impact on people's behavior specifically environmentally related products including BEVs (White and Sintov, 2017; Vongurai, 2020). It can be noted that environmental behavior was very predictive when purchasing BEVs as public environmental awareness, and concerns toward negative outcomes of global warming and climate change is increasing. The customer perceived value of BEV as a green product therefore will positively and significantly predict intention to adopt BEVs in Malaysia.

This finding is also supported in the United States, which ecological awareness and environmental concern are shown to have a positive and significant influence on intention to purchase fuel-efficient vehicles and electric cars (Gallagher and Muehlegger, 2011). Moreover, customers' positive attitudes towards using electric cars as environmentally friendly vehicles have been proven in many studies (Moons and De Pelsmacker, 2012; Egbue and Long, 2012; Egbue and Long, 2012, 2012; Carley et al., 2013). Another study in Hong Kong showed that society comprehends the environmental benefit of electric cars but not the social and economic benefits (Delang and Cheng, 2013).

Environmental concerns and customer awareness of human effects on environmental problems have been speculated to affect the buying intention of electric cars (Egbue and Long, 2012; Carley et al., 2013). Although consumers stated doubt about the positive environmental effects of electric cars in some exploratory research (Caperello and Kurani, 2011).

In studies on the acceptance of environmentally friendly technologies, the environmental concerns and norms are theorized to influence the intentions of purchasers (Rezvani et al., 2018). In Value Belief Norm theory, Stern (2000) argues that personal principles are inspired by norms and environmental awareness. Moreover, their conduct is also shaped through the chain of personal values, associated with ecological concerns.

Therefore, this finding is particularly important for the Malaysian government. The results support fact that individual's awareness and concerns about environmental issues leads to consumer acceptance of BEVs in Malaysia.

For commercial purposes, the dealers are expected to create more educational promotions to alert the consumers on the advantages and importance of BEVs for having cleaner air specifically in daily routine usage. The government should play the role to increase public awareness about the thread of conventional transportation as the main sector identified to produce carbon dioxide emissions in the country. Besides tax exemption, the government should more campaigns to make individuals aware about what the benefit of electric vehicles to the environment.

### 11.6 Moderating effect of personal innovativeness

The results of moderation analysis indicated a significant moderation effect of personal innovativeness on the correlation between social influence and willingness to purchase BEVs. Moreover, from the conditional effects of values of the moderator, the moderation effect is significant in both lower and higher levels. The findings are in line with Rogers (2003). He argued that only people with high innovativeness are intended to buy a new technology at its initial stage. Agarwal and Prasad (1998) also argued that those individuals with a greater level of innovativeness are speculated to be more intendeds to buy the new technologies than those with a lower level.

Consumer innovativeness, according to Rogers (2003), is the degree to which an individual adopts new ideas earlier than the average member of his or her social class. Because new products are critical to the survival of many businesses, the dissemination of innovation has been extremely significant in marketing and customer behavior. In general, customers with a high level of innovativeness can be identified by: their openness to change concepts and products and their ability to persuade people to adopt creative ideas and products. A person with high innovativeness has also the ability to address the problems and make faster buying decisions when there are dramatic changes in the market (Rogers, 2003). Prior research suggests that innovative customers frequently provide information and suggestions about new items to other consumers and that their opinions are generally accepted and influenced by other consumers. The diffusion of innovation theory (Rogers, 1995) explains how novel items move through social systems when they are embraced or rejected by individuals. Interindividual differences in how people react to new things are explained as innovativeness, and it accounts for most of their success or failure.

As a result, innovators may progressively adopt BEVs, while laggards adopting them slowly or never at all. As a result, measuring inventiveness as moderation is an important activity for both theory testing and practical purposes as demand for BEVs grows. Several studies have already stressed the significance and prominence of social norms among Malaysians (Onel, 2017; Hasbullah et al., 2016; Sin et al., 2012). As a result, the findings of this study show that consumer innovativeness influences consumer choice for Battery Electric Vehicles (BEVs) through regulating the relationship between social influence and purchase intention. This is an important contribution of the research is the moderation effect of personal innovativeness on the influence of social norms. Consistent with the diffusion of innovation theory, this finding will reveal that those who have higher personal innovativeness traits, will be more likely to ignore the social norms and adopt BEVs. This finding will be beneficial for dealers and officials to target early adopters with higher innovative characteristics. It is, therefore, crucial to recognize people with a high level of innovativeness, as they are at ease with taking risks. They are informed that the new product might not deliver all the advantages that they expect yet are enthusiastic to be in the first line of adoption.

### 11.7 Moderating effect of driving experience

The proposed moderation model shows that the driving experience does not have a moderation impact on the association between social influence and intention to use BEV. From the conditional effects of values of the moderator, the moderation effect was not significant. The results show that the P-value of interaction is 0.2943 which is more than 0.05. The findings also indicate that driving experience is not a moderator in relationship facilitating conditions and willingness to use BEVs. The P-value of interaction is 0.0597 which is more than 0.05 and the confidence interval includes zero. Nevertheless, the study showed that diving experience considerably moderates association among range anxiety and intention to use. The positive high interaction coefficient (β = 0.7537) suggests that driving a BEV will have a positive moderating effect on the correlation between range anxiety and intention to use them. It means that the more driving experience is, the more positive becomes the effect of range anxiety on intention to use electric cars. In other words, driving electric vehicles will reduce the negative impact of range anxiety on the acceptance of electric cars.

Although the construct “experience” was widely used in information technology acceptance research, at the time when the study was conducted, very few prior studies tested the moderation effect of “driving experience” on the relationship between range anxiety and intention to use. According to the results of this study, driving experience helps customers to better know the way a BEV works, and this will shift their previous perceptions or fear of driving range. They may think more optimistically, particularly when they feel the pleasure of experiencing less noise, smoothness along with high performance and acceleration. Moreover, even one driving experience might facilitate their understanding that electric cars are as practical as conventional cars specifically in short or routine journeys. Therefore, one of the important implications of the study is that the negative effect of range anxiety will be significantly moderated by the experience of driving the vehicle, by potential customers. According to this finding which will be significant for car dealers, even driving a BEV once, may decrease their range anxiety and encourage the potential buyer to use them.

Some of the other factors that affect customer's preferences are low cost of maintenance, reduced air pollution, and better vehicle performance, gas price, and performance (Lieven et al., 2011; Egbue and Long, 2012; Carley et al., 2013; Jensen et al., 2013; Krupa et al., 2014; Zhang et al., 2011; Moons and De Pelsmacker, 2012). Some research recommended financial incentives and public policies (Gallagher and Muehlegger, 2011) energy and price (Jensen et al., 2013) as predictors of EVs adoption.

### 11.8 Implications of the study

The study has important implications. Some of the key practical and theoretical implications are:

This study evolved a technology acceptance framework through developing the UTAUT2 model by adding important factors influencing electric cars according to previous studies such as range anxiety, personal innovativeness, driving experience, and environmental concern, to study the acceptance of the BEV technology in Malaysia. Specifically, this study has contributed moderating influence of driving experience and personal innovativeness to the technology acceptance literature in the automotive context. The findings are significant to electric car producers and policymakers who have environmental concerns to understand consumer perspectives toward the usage of BEVs.

According to data analysis, respondents' environmental concern was the most important predictor of BEV acceptance. Therefore, prospective purchasers believe that BEVS has a constructive impact on ecological protection by less polluting the environment. Consequently, it can be recommended to promote a green lifestyle. BEV producers might advertise the premise of their environmental benefits to enhance users’ awareness of their advantages and intentions to buy them.

Potential customers admire the hedonic feeling of driving BEV as a quiet vehicle with smooth acceleration. It means they will be satisfied with the unique attributes, performance, and efficacy of BEVs. Hedonic motivation was found to be one the most important factors affecting their intentions to use them.

Respondents of this study also expressed concerns about the lack of technical supports necessary for using BEVs in Malaysia. Most importantly constrained driving range, after-sale support, and insufficient charging infrastructure. This suggests that expanding the driving range and supplying sufficient charging stations will decrease concerns about driving a BEV, which respondents hold.

Another important contribution of the research is the moderation effect of personal innovativeness on the influence of social norms. Consistent with the diffusion of innovation theory, this finding reveals that those with higher personal innovativeness traits will be more likely to ignore the social norms and adopt BEVs. This finding will be beneficial for dealers and officials to target early adopters with higher innovative characteristics. It is, therefore, crucial to recognize people who are at ease with taking risks. Therefore, scientists, entrepreneurs, academicians, and technology enthusiasts can be targeted as the first line of adoption.

More importantly, the results show that the driving experience of BEVs will significantly decrease the potential consumers' concerns about the limited range of the vehicle which is a significant implication for dealers and manufacturers. This is an important implication of the study is that the negative effect of range anxiety will be significantly moderated by the experience of driving the vehicle. Therefore, it is recommended that dealers and manufacturers provide opportunities for the public and encourage them to test the electric vehicles, as that will improve the potential users’ perception, and eventually will increase the purchasing intentions. Moreover, driving experience might facilitate their understanding that electric cars are more efficient and practical in short or routine rides.

### 11.9 Limitations of the study

There are some limitations to this study. Because of the lack of time and resources, along with complexity in evaluating the intention, the current study surveyed one university campus together with five different companies. Therefore, the sampling method used in this study aimed to survey postgraduates, University lecturers, and managers of those technology companies who were willing to participate (as early adopters), and consequently the sample of this study does not confirm that subdivision represents the entire population in Malaysia. Moreover, some of the respondents might not experience or even observed BEVs, and this might limit the strength of their responses. Finally, another limitation with this study is that demographic factors were not considered for evaluation. Further studies may examine the impact of various demographic characteristics on the adoption of BEVs in Malaysia.

This study did not include some of the constructs of the UTAUT 2 model such as performance expectancy, habit, and price value. Future investigations may include these factors to test the acceptance of BEVs in Malaysia. Moreover, the sample of the study is limited in different areas of Kuala Lumpur. Therefore, the results of the study might not be generalizable for the whole Malaysian society.

### 11.10 Conclusion

This study aimed to determine the correlations among social influence, facilitation condition, environmental concern, range anxiety, and perceived enjoyment on intention to purchase battery electric vehicles in Malaysia. The current study proposed a novel conceptual framework to explain and predict battery electric vehicle adoption. The results of this research indicated that the developed model provides a good fit for all the constructs used in the current research. The outcome showed that social influence, facilitation condition, environmental concern, and perceived enjoyment all have a positive and significant effect on the intention to use BEVs. At the same time, range anxiety had a negative effect on intention as expected. Remarkably, this study indicates that the driving experience of electric cars has a moderation influence on the relationship between anxiety and BEV adoption. Moreover, personal innovativeness moderates the correlation between social influence and intention of use of BEVs.

## Declarations

### Author contribution statement

Hamed Khazaei: Conceived and designed the experiments; Performed the experiments; ​Analyzed ​and interpreted the data; Contributed reagents, materials, analysis tools or data; Wrote the paper.

Mohammad Ali Tareq: Conceived and designed the experiments; Contributed reagents, materials, analysis tools or data.

### Funding statement

This research did not receive any specific grant from funding agencies in the public, commercial, or not-for-profit sectors.

### Data availability statement

Data associated with this study has been deposited at ​Data in Brief at https://www.sciencedirect.com/science/article/pii/S2352340919309990?via%3Dihub.

### Declaration of interests statement

The authors declare no conflict of interest.

### Additional information

No additional information is available for this paper.
